# Reducing the global burden of cerebral venous thrombosis: An international research agenda

**DOI:** 10.1177/17474930241242266

**Published:** 2024-04-09

**Authors:** Jonathan M Coutinho, Anita van de Munckhof, Diana Aguiar de Sousa, Sven Poli, Sanjith Aaron, Antonio Arauz, Adriana B Conforto, Katarzyna Krzywicka, Sini Hiltunen, Erik Lindgren, Mayte Sánchez van Kammen, Liqi Shu, Tamam Bakchoul, Rosalie Belder, René van den Berg, Elisheva Boumans, Suzanne Cannegieter, Vanessa Cano-Nigenda, Thalia S Field, Isabel Fragata, Mirjam R Heldner, María Hernández-Pérez, Frederikus A Klok, Ronen R Leker, Lia Lucas-Neto, Jeremy Molad, Thanh N Nguyen, Dirk-Jan Saaltink, Gustavo Saposnik, Pankaj Sharma, Jan Stam, Vincent Thijs, Michiel van der Vaart, David J Werring, Diana Wong Ramos, Shadi Yaghi, Nilüfer Yeşilot, Turgut Tatlisumak, Jukka Putaala, Katarina Jood, Marcel Arnold, José M Ferro

**Affiliations:** 1 Department of Neurology, Amsterdam UMC, location University of Amsterdam, Amsterdam, The Netherlands; 2 Stroke Center, Centro Hospitalar Universitário Lisboa Central, Institute of Anatomy, Faculdade de Medicina, Universidade de Lisboa, and L Lopes Lab, Instituto de Medicina Molecular JLA, Lisbon, Portugal; 3 Department of Neurology & Stroke, University of Tübingen, Tübingen, Germany; 4 Hertie Institute for Clinical Brain Research, University of Tübingen, Tübingen, Germany; 5 Christian Medical College Hospital, Vellore, India; 6 Instituto Nacional de Neurologia y Neurocirugia Manuel Velasco Suarez, Mexico City, Mexico; 7 LIM-44, Hospital das Clinicas HCFMUSP, Faculdade de Medicina, Universidade de Sao Paulo, Sao Paulo, Brazil; 8 Helsinki University Hospital and University of Helsinki, Helsinki, Finland; 9 Department of Neurology, Sahlgrenska University Hospital and Department of Clinical Neuroscience, Institute of Neuroscience and Physiology, Sahlgrenska Academy at University of Gothenburg, Gothenburg, Sweden; 10 Brown University, Providence, RI, USA; 11 Centre for Clinical Transfusion Medicine, Medical Faculty of Tübingen, University of Tübingen, Tübingen, Germany; 12 Netherlands Thrombosis Foundation, Voorschoten, The Netherlands; 13 Department of Radiology and Nuclear Medicine, Amsterdam UMC, location University of Amsterdam, Amsterdam, The Netherlands; 14 Patients’ Representative, The Netherlands; 15 Department of Clinical Epidemiology, Leiden University Medical Center, Leiden, The Netherlands; 16 Vancouver Stroke Program, Division of Neurology, University of British Columbia, Vancouver, BC, Canada; 17 NOVA Medical School, Universidade NOVA de Lisboa, Lisboa, Portugal; 18 Inselspital Bern, University Hospital and University of Bern, Bern, Switzerland; 19 Hospital Germans Trias i Pujol, Badalona, Spain; 20 Department of Medicine—Thrombosis and Hemostasis, Leiden University Medical Center, Leiden, The Netherlands; 21 Hadassah—Hebrew University Medical Center, Jerusalem, Israel; 22 North Lisbon University Hospital Center and Lisbon Medical School, Lisbon, Portugal; 23 Tel Aviv Medical Center, Tel Aviv, Israel; 24 Boston Medical Center, Boston, MA, United States; 25 Dutch Brain Foundation, The Hague, The Netherlands; 26 Stroke Outcomes & Decision Neuroscience Research Unit, University of Toronto, Toronto, ON, Canada; 27 Royal Holloway University of London, London, United Kingdom; 28 Florey Institute of Neuroscience and Mental Health, Parkville, VIC, Australia; 29 Department of Medicine, The University of Melbourne, Parkville, VIC, Australia; 30 Dutch Heart Foundation, The Hague, The Netherlands; 31 UCL Queen Square Institute of Neurology, London, United Kingdom; 32 Portugal AVC-União de Sobreviventes, Familiares e Amigos, Portugal; 33 Istanbul Faculty of Medicine, Istanbul University, Istanbul, Turkey; 34 Hospital da Luz, University of Lisbon, Lisbon, Portugal

**Keywords:** CVT, thrombosis, epidemiology, pathophysiology, treatment, summit, international

## Abstract

**Background:**

Due to the rarity of cerebral venous thrombosis (CVT), performing high-quality scientific research in this field is challenging. Providing answers to unresolved research questions will improve prevention, diagnosis, and treatment, and ultimately translate to a better outcome of patients with CVT. We present an international research agenda, in which the most important research questions in the field of CVT are prioritized.

**Aims:**

This research agenda has three distinct goals: (1) to provide inspiration and focus to research on CVT for the coming years, (2) to reinforce international collaboration, and (3) to facilitate the acquisition of research funding.

**Summary of review:**

This international research agenda is the result of a research summit organized by the International Cerebral Venous Thrombosis Consortium in Amsterdam, the Netherlands, in June 2023. The summit brought together 45 participants from 15 countries including clinical researchers from various disciplines, patients who previously suffered from CVT, and delegates from industry and non-profit funding organizations. The research agenda is categorized into six pre-specified themes: (1) epidemiology and clinical features, (2) life after CVT, (3) neuroimaging and diagnosis, (4) pathophysiology, (5) medical treatment, and (6) endovascular treatment. For each theme, we present two to four research questions, followed by a brief substantiation per question. The research questions were prioritized by the participants of the summit through consensus discussion.

**Conclusions:**

This international research agenda provides an overview of the most burning research questions on CVT. Answering these questions will advance our understanding and management of CVT, which will ultimately lead to improved outcomes for CVT patients worldwide.

## Introduction

Cerebral venous thrombosis (CVT) is an uncommon cause of stroke that mainly affects young adults. The overall incidence of CVT is between 1.2 and 1.6 per 100,000 person-years,^1,2^ but the incidence is on the rise and varies considerably between regions in the world.^2^ Women are up to three times more frequently affected than men, due to the fact that female hormones are an important risk factor for CVT.^3^ Severe headache, present in about 90% of patients, is the most common symptom. Approximately 50% to 60% of patients with CVT develop a brain parenchymal lesion (most often brain edema or intracerebral hemorrhage), which can lead to focal neurological deficits, epileptic seizures, and coma.^4^ Heparin followed by oral anticoagulation, generally for a period of 3 to 12 months, is the main treatment of CVT and is recommended by all major international guidelines.^5,6^ The mortality of CVT has decreased substantially over time and is now between 3–15%.^7,8^ While most surviving patients do not have major physical disability, chronic debilitating symptoms such as headache, fatigue, neurocognitive deficits, and epileptic seizures frequently continue to affect patients in their activities of daily living, and often result in a long-term diminished quality of life.^9–11^

Due to the rarity of CVT, performing high-quality scientific research in this field remains a challenge. Important advancements have been gained over the years, generally by virtue of international collaborations. Nevertheless, many areas of uncertainty remain. In addition, present knowledge about CVT is mostly based on studies from high- and middle-income countries, while data from low-income countries are sparse. Providing answers to unresolved research questions could improve prevention, diagnosis, and treatment and ultimately translate to a better outcome of patients with CVT.

In June 2023, the International Cerebral Venous Thrombosis Consortium organized a research summit on CVT in Amsterdam, the Netherlands (Supplemental Figure 1 available online). The International Cerebral Venous Thrombosis Consortium^12^ is a scientific collaboration currently involving 117 CVT experts from 29 countries across five continents. The summit brought together 45 invited participants from 15 countries and consisted of clinical researchers from various disciplines (both from within and outside of the consortium), patients who previously suffered a CVT, and delegates from industry and non-profit funding organizations (full participant list in Supplemental Table 1 available online). The overarching aim of the CVT summit was to formulate an international research agenda, in which the most important research questions in the field of CVT are prioritized. During this 2-day event, six pre-specified themes were discussed: (1) epidemiology and clinical features, (2) life after CVT, (3) neuroimaging and diagnosis, (4) pathophysiology, (5) medical treatment, and (6) endovascular treatment. The current article presents the research agenda for each of these themes that was agreed upon by the attendees. By publishing this research agenda, we aim to achieve three distinct goals: (1) to provide inspiration and focus to research on CVT for the coming years, (2) to further reinforce international collaboration in the field of CVT, and (3) to facilitate the acquisition of funding for future studies with which the questions of this agenda could be answered.

## Methods

**Figure 1.**
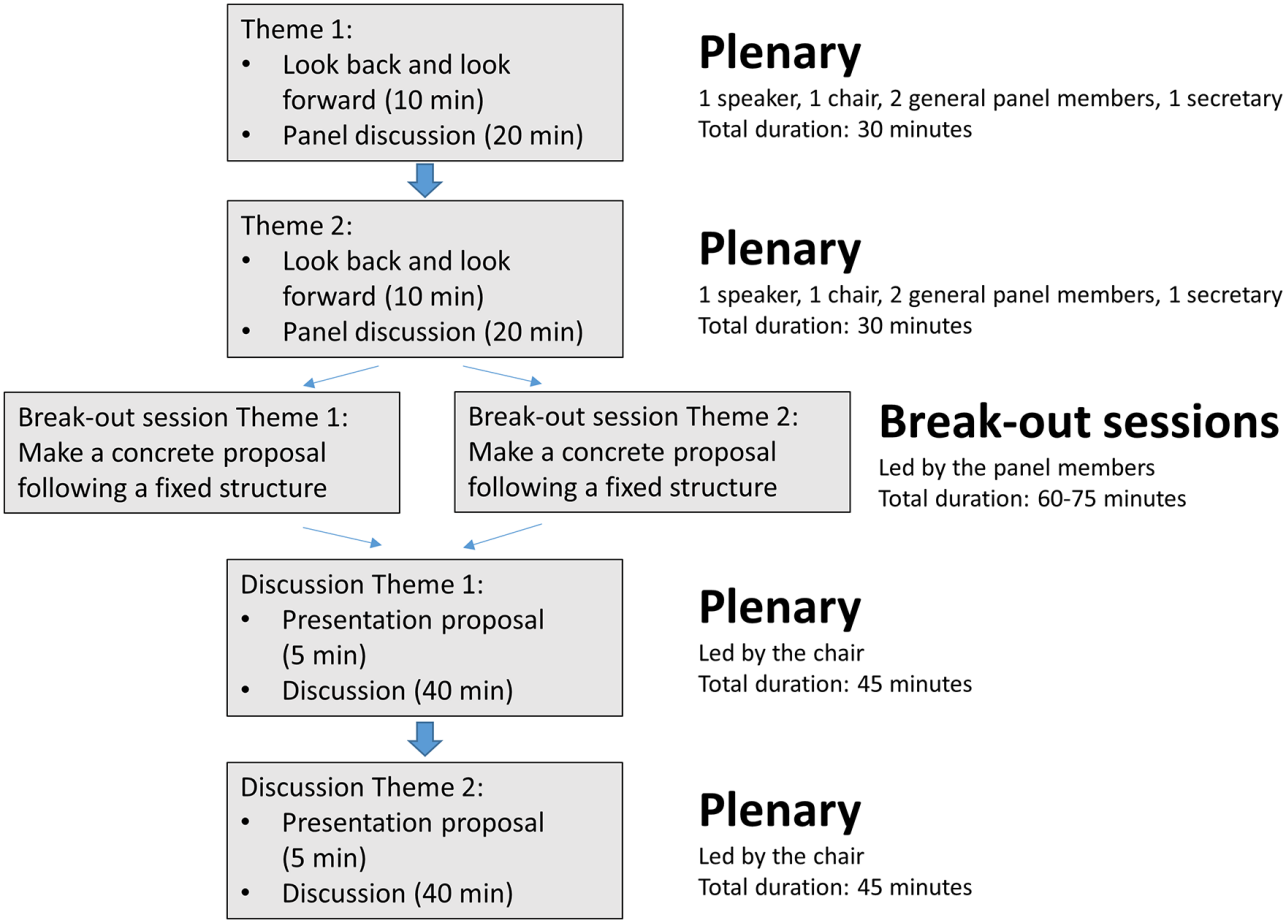
Structure of the meeting sessions.

During the summit, each theme was introduced by a speaker who presented the current state of knowledge, summarized ongoing research activities, and proposed topics for future research. After this presentation, the meeting participants, led by panel members with specific expertise in that particular research area (Supplemental Table 2 available online), discussed the theme and provided input for the research agenda. Each time after discussing two themes in a plenary session, the participants were split into two breakout groups (one for each theme). Each breakout group continued working on their theme, constructing a selected list of concrete research questions in order of importance. Thereafter, all participants conveyed in a second plenary session to discuss the research questions proposed by the breakout groups. Following this discussion, the research questions were finalized. This resulted in a limited number of research questions per theme (Table 1). All participants agreed—by means of consensus discussion—that these questions should be the focus of research in the coming years. No formal voting was held to order the research questions based on priority. The structure of the meeting is depicted in Figure 1. The research agenda is categorized according to the six themes. For each theme, we present the prioritized research questions, followed by a brief substantiation per question.

**Table 1.** Overview of research questions per theme.

**Theme**	**Research questions**
Epidemiology and clinical features	1. *What are the global trends in incidence and regional variations in risk factors and clinical manifestations of CVT?* 2. *Should patients with CVT be screened for occult cancer?*
Life after CVT	1. *How can we best measure recovery and residual symptoms after CVT?* 2. *What are the very long-term outcomes of patients with CVT?* 3. *Does structured patient counseling after CVT lower the burden of long-term sequelae and improve quality of life?*
Neuroimaging and diagnosis	1. *Can clinical scores and artificial intelligence-based algorithms improve diagnosis and ruling out of CVT?* 2. *What is the optimal modality, timing, and grading method to assess recanalization of thrombosed veins/sinuses after CVT?* 3. *What are early clinical or imaging predictors of neurological deterioration and/or poor functional outcome in CVT patients?*
Pathophysiology	1. *What is the pathophysiology of thrombosis in CVT and how does it differ from other locations of thrombosis?* 2. *Which inflammatory and coagulation factors underlie the cerebral response and tissue damage in CVT?* 3. *Which genetic variants are associated with CVT?*
Treatment—medication	1. *Do direct oral anticoagulants (DOACs) have non-inferior efficacy and superior safety over standard anticoagulation and does long-term treatment with reduced-dose DOACs prevent recurrent thrombosis in patients with a high risk of recurrence?* 2. *Does anti-edema therapy with anti-inflammatory drugs improve outcomes of patients with CVT?* 3. *Does prophylactic or prolonged treatment with anti-epileptic drugs (AED) prevent seizures and improve quality of life in patients at high risk of remote seizures?* 4. *Does treatment of intracranial hypertension in the acute phase of CVT reduce headache and improve long-term outcomes of patients with CVT?*
Treatment—endovascular	1. *Is there a subgroup of CVT patients who may benefit from endovascular treatment?* 2. *What is the best endovascular approach to achieve fast and safe recanalization in CVT?*

## Research questions

### Theme 1—epidemiology and clinical features

#### What are the global trends in incidence and regional variations in risk factors and clinical manifestations of CVT?

Rationale: The incidence of CVT is increasing and is thought to be higher in low- and middle-income countries than in high-income countries.^2,13–15^ Etiologies may differ according to age, sex, genetics, climate conditions, altitude, exposure to pollutants, socioeconomic circumstances, and lifestyle, and may also partly overlap with other types of venous thromboembolisms. CVT is historically more common among women than men, but recent small-scale studies suggest that this ratio is shifting to a more balanced sex ratio.^13^ The causal mechanisms for the trends and regional variation in epidemiology are poorly understood.^4,16^ The clinical presentation of CVT is highly heterogeneous—ranging from isolated chronic headache to acute focal neurological deficits, seizures, and coma with irreversible parenchymal injury.^4,17,18^ A few distinct clusters of clinical syndromes, possibly warranting specific management, have been suggested including isolated intracranial hypertension, focal neurological syndrome, and diffuse encephalopathy.^19^ However, evidence on how to approach these specific subgroups is limited, and the clinical relevance of these subgroups is not fully understood. It is unknown if clinical symptoms of CVT vary between different regions. Current data on risk factors and clinical symptoms in CVT mostly come from cohort studies performed in high-income countries. Large international case–control studies—with particular focus on low- and middle-income countries—are needed to close these knowledge gaps.

#### Should patients with CVT be screened for occult cancer?

Rationale: Active cancer, both solid and hematological, is a common risk factor for CVT and is present in around 7–15% of patients with CVT at the time of diagnosis.^4,16,20,21^ Among patients with CVT aged 55 years and older, cancer is present in a quarter of the patients.^17^ Recent studies found that the risk of occult cancer, that is, cancer diagnosed after CVT occurred, may be increased after a first episode of CVT.^22,23^ However, these studies were underpowered and had conflicting results. Moreover, it is unknown if screening for occult cancer is warranted, and if so, in which patients and with which diagnostic techniques. Current guidelines do not recommend routine screening for occult malignancy and call for further research.^5,6^ There is a need for larger studies that assess the risk of occult cancer after CVT, identify subgroups at particular risk, and explore the (cost-)effectiveness of screening for occult cancer after CVT.

### Theme 2: life after CVT

#### How can we best measure recovery and residual symptoms after CVT?

Rationale: Compared with arterial ischemic stroke, patients with CVT are much younger and their residual deficits more often consist of cognitive rather than physical impairments. As a result, commonly used stroke outcome scales, like the modified Rankin Scale, seem to be less suitable to assess functional recovery after CVT given that an outcome of functional independence may not reflect the high burden of residual symptoms that survivors may experience.^11,24^ Cohort studies that followed up patients for a long period found that cognitive problems (40–70%), fatigue (30–40%), chronic headache (20–40%), and depression (20–35%)^9,10,25^ frequently affect patients with CVT and that these symptoms negatively impact their daily activities and quality of life. One study, for instance, found that approximately 30% of patients who have suffered from CVT were unable to resume their previous work.^25^ There is a need for developing and validating a CVT outcome scale that captures the aforementioned elements, as well as other important domains including return to work and work-related coping, study, family life, and quality of life. Patient reported outcome measures (PROMs) may be suitable for this purpose, but require further investigation.^26,27^ In addition to a dedicated instrument to capture outcomes of CVT, development of a standardized minimum set of outcomes and outcome measurements will further facilitate integration of patient-relevant PROMS into both clinical practice and clinical trials. This set can be used to guide clinical decision-making and benchmarking the quality of care.

#### What are the very long-term outcomes of patients with CVT?

Rationale: Residual symptoms appear to be very common after CVT and are reported in up to 70% of patients.^9,10^ Most cohort studies had a follow-up of 1 to 3 years,^4,28^ which, given the young age of patients with CVT, is probably not long enough to capture the chronic state of the patients and trajectory of residual symptoms. Some CVT-related problems may not arise until years after initial diagnosis, such as thrombotic recurrences, late seizures, dural arteriovenous fistulas, and pregnancy-related complications. On the contrary, some residual symptoms may resolve over time. There is thus a need for large, multicenter, prospective cohort studies in which patients with CVT are followed up for a longer period, ideally at least 5–10 years.

#### Does structured patient counseling after CVT lower the burden of long-term sequelae and improve quality of life?

Rationale: Information for patients with CVT is available from many sources. However, no (international) patient information platform with simple and structured information about CVT and possible long-term residual symptoms is available. A patient information platform may improve awareness of residual symptoms after CVT and thereby could lower the burden of long-term sequelae. This platform could, for example, include a standardized checklist of symptoms, interactive and informative videos, and a social network forum to get in touch with other patients who have had CVT. This could support and enhance informal patient groups already in place in various countries. Effectiveness of this strategy on patient outcomes could be compared with standard care in a controlled setting or could be evaluated using questionnaires.

### Theme 3: neuroimaging and diagnosis

#### Can clinical scores and artificial intelligence-based algorithms improve diagnosis and ruling out of CVT?

Rationale: Due to the heterogeneity of its clinical and radiological presentation, accurately diagnosing CVT in the acute setting remains challenging. Early diagnosis is important because earlier treatment can prevent potential complications like brain parenchymal lesions. A diagnostic tool aiding in the detection of CVT on non-contrast-enhanced computed tomography (CT), which is often the initial imaging performed in patients with acute neurologic symptoms, could help to improve diagnostic accuracy and decrease time to diagnosis. This can be achieved through automated detection of, for example, hyper-attenuated venous sinuses, focal brain edema, or juxtacortical bleeding.^29^ As exemplified in acute ischemic stroke, artificial intelligence-based diagnostic tools allow for faster and more accurate diagnosis resulting in improvement of workflow efficiency,^30–32^ and are already successfully used in clinical practice.^33,34^ In a recent study, a deep learning algorithm improved the detection of CVT on routine magnetic resonance (MR) imaging and identified up to 44% more thrombosed segments than radiologists, underlining a high potential of this approach.^35^ On the contrary, only 1 out of 10 patients in whom neuroimaging is performed because of suspected CVT turn out to have CVT.^36,37^ Given this low yield, there is room for improved selection of patients for specific diagnostic imaging, especially in low- and middle-income countries where resources are generally more sparse. The use of a risk-stratifying clinical score in combination with a biomarker similar to what is achieved in the diagnosis of pulmonary embolism using a clinical decision rule and a D-dimer test can lead up to a 30–50% reduction in imaging and significant improvement of workflow.^38–40^ A recently developed clinical score combined with a D-dimer value shows promising performance in selecting patients at risk of CVT (negative predictive value of 94%), but this approach requires validation prior to implementation.^41^

#### What is the optimal modality, timing, and grading method to assess recanalization of thrombosed veins/sinuses after CVT?

Rationale: Recanalization, particularly in the early stages, is associated with the regression of non-hemorrhagic parenchymal lesions, reduced brain tissue damage, a better functional outcome, and a lower risk of recurrence.^42–48^ Therefore, gaining understanding of temporal patterns of recanalization is important. In addition, early recanalization may serve as a surrogate endpoint for new therapies. However, current imaging practices differ: imaging is performed at varying time points, using non-standardized imaging techniques, and there is no uniform grading scale to assess recanalization.^42–44^ Thrombus imaging, including black blood technique, and quantification of the arteriovenous transit time of contrast using time-resolved 4D-MR-angiography or 4D-CT-angiography can be employed to study radiological thrombus properties, quantify thrombus load, and describe specific flow patterns.^49–53^ This information is expected to help guide treatment decisions and predict long-term outcomes, including the presence of residual symptoms. However, availability and cost-effectiveness of these imaging techniques should be studied.

#### What are early clinical or imaging predictors of neurological deterioration and/or poor functional outcome in CVT patients?

Rationale: Early identification of patients with CVT at high risk of deterioration or poor clinical outcome is instrumental to guide treatment decisions and the design of therapeutic trials. Clinical factors associated with poor outcome in two recently published prognostic CVT scores included neurological deficit, presence of coma on presentation, active cancer, and decreased hemoglobin level.^54,55^ On neuroimaging, the presence of intracranial hemorrhage was associated with poor outcome in both the IN-ReVASC and SI_2_NCAL_2_C prognostic scores after CVT.^54,55^ With advancement in imaging techniques, there are growing opportunities to study novel imaging predictors of clinical outcome.^54,56–59^ For example, incorporating CT-perfusion imaging, net water uptake measurements, and dynamic (4D) MR-angiography techniques can help to classify and predict the temporal and spatial patterns of parenchymal lesions.^60,61^ Other potential indicators include specific location and extent of the thrombosis, brain tissue damage markers, poor venous collateral circulation, and lack of early recanalization. Identification of early predictors for clinical deterioration or poor functional outcome after CVT would facilitate the development of personalized therapeutic strategies, improve patient counseling, and optimize the selection of patients for future clinical trials, for instance regarding endovascular treatment or new thrombolytic or neuroprotective drugs.

### Theme 4: pathophysiology

#### What is the pathophysiology of thrombosis in CVT and how does it differ from other locations of thrombosis?

Rationale: Very little is known about the pathophysiology of CVT. Most of the previous research has focused on hypercoagulability, but the role of endothelial dysfunction—which is known to play a pivotal role in thrombosis—has largely been neglected. Cerebral endothelium as part of the blood–brain barrier has unique features when compared to endothelium in other organ systems, among others the rich presence of tight junctions and lack of valves in the venous system. Ex vivo models of thrombosis have made it possible to study endothelium–blood interactions in the development of venous thrombosis with increasing detail.^62^ Studies with “thrombosis-on-a-chip” models^62^ of the cerebral venous endothelium could shed light on how the pathogenesis of CVT differs from that of thrombosis at other sites. In addition, analysis of cerebral thrombi, for instance collected during endovascular thrombectomy, may provide more insight into underlying pathophysiological processes. A better understanding of the pathophysiology of thrombosis in the intracranial venous system could also help to identify new treatment options, including novel antithrombotic drugs.

#### Which inflammatory and coagulation factors underlie the cerebral response and tissue damage in CVT?

Rationale: The inflammatory reaction of the brain in response to CVT is poorly understood.^63^ Inflammation and perihematomal edema are emerging as key targets for treatment of spontaneous intracerebral hemorrhage^64^ and may also be relevant to brain injury and outcome after CVT. For example, inflammatory cytokines or nuclear factor erythroid 2-related factor 2 (NRF-2) provide relevant pathways and treatment targets in intracerebral hemorrhage.^65^ Modulation of the central nervous system inflammatory response has also been shown to improve outcome in bacterial meningitis^66^ and reduce symptoms caused by edema in brain tumors,^67^ although no effective modulatory agents have been identified to date in other diseases such as ischemic stroke or traumatic brain injury.^68,69^ Identification of biochemical markers associated with tissue damage in patients with CVT would be the first step toward isolating potential targets for therapeutic intervention. A large international biobank containing blood samples from patients with CVT and healthy controls from as many countries as possible could provide the means to systematically study markers of inflammation associated with CVT in matched case–control studies. Analogous to *The Multiple Environmental and Genetic Assessment of risk factors for venous thrombosis study* (MEGA study) in venous thromboembolism,^70^ such a study could combine clinical information with biobank data at standardized time points encompassing the acute to chronic phase and thus answer multiple research questions embedded within a single clinical study.

#### Which genetic variants are associated with CVT?

Rationale: The aforementioned clinical study with biobank data could be further extended to include a large genome-wide association study with adequate representation of non-European populations. The first genome-wide association study of CVT included 882 patients and 1205 healthy controls and revealed an association of the ABO blood group gene with the development of CVT.^71^ With larger sample sizes, other genetic variants associated with CVT occurrence, outcome after CVT, and response to certain medications could be identified.

### Theme 5: treatment—medication

#### Do direct oral anticoagulants (DOACs) have non-inferior efficacy and superior safety over standard anticoagulation and does long-term treatment with reduced-dose DOACs prevent recurrent thrombosis in patients with a high risk of recurrence?

Rationale: Current guidelines recommend treating CVT with low molecular weight heparin followed by oral anticoagulation.^5,6^ Vitamin K antagonists (VKAs) and, increasingly, direct oral anticoagulants (DOACs) are the preferred types of oral anticoagulants.^72^ The evidence underlying the use of DOACs in CVT, however, comes from two small randomized trials and one retrospective multicenter study.^11,28,73^ Large real-world data sets with prospectively collected and adjudicated endpoints are required to confirm the efficacy and safety of DOACs for treatment of CVT. Moreover, DOACs are now frequently being used in a reduced dose for long-term treatment after unprovoked venous thromboembolism (VTE) because they effectively prevent recurrent thrombotic events with a minimal risk of bleeding.^74,75^ To maximize the benefits and minimize the risks, patients should be carefully selected based on their risk of recurrent thrombotic events.^74^ The thrombotic recurrence rate after CVT is generally lower than after VTE,^4,20^ but it is unknown what the long-term recurrence rate is in specific subgroups of patients, such as patients with unprovoked thrombosis or persistent risk factors. If these patients have a sufficiently high risk of thrombotic recurrence, a clinical trial that examines the efficacy and safety of prolonged treatment with reduced-dose DOACs would be justified. Furthermore, new types of anticoagulant or fibrinolytic drugs such as Factor XIa inhibitors and alpha-2-antiplasmin inhibitors are promising and are currently being tested in the treatment of VTE and stroke.^76–81^ In patients with CVT, these drugs may have the potential to improve early recanalization, reduce bleeding events, and improve functional recovery.^9,42^ The efficacy and safety of these novel anticoagulation and fibrinolytic drugs need to be evaluated in comparison with standard treatment.

#### Does anti-edema therapy with anti-inflammatory drugs improve outcomes of patients with CVT?

Rationale: Brain edema is a frequent complication of CVT and is generally vasogenic in origin.^82,83^ Anti-edema drugs such as steroids or anti-inflammatory toll-like receptor 4 antagonists could potentially reduce formation of edema,^84,85^ thereby reducing the risk of seizures and transtentorial herniation, and thus improve patient outcome. On the contrary, steroids can also induce a pro-thrombotic state and are associated with an increased risk of VTE.^86^ A previous non-randomized cohort study on the efficacy of steroids in acute CVT showed no benefit of steroid use and even harm in patients without parenchymal lesions,^87^ but evidence from prospective, controlled studies of anti-inflammatory drugs is lacking.

#### Does prophylactic or prolonged treatment with anti-epileptic drugs (AED) prevent seizures and improve quality of life in patients at high risk of remote seizures?

Rationale: Late seizures occur in approximately 10% of patients after CVT and often have a substantial impact on activities of daily living and quality of life.^88,89^ Multiple risk factors for late seizures have been identified, such as intracranial hemorrhage or symptomatic seizures in the acute phase of CVT.^88,90^ Assessment tools to identify patients at high risk of late seizures are currently being developed. A study is needed to assess whether prophylactic or prolonged treatment with anti-seizure medication or epileptogenesis-inhibiting agents in high-risk patients prevents late seizures and improves quality of life.

#### Does treatment of intracranial hypertension in the acute phase of CVT reduce headache and improve long-term outcomes of patients with CVT?

Rationale: Intracranial hypertension affects around 80% of patients in the acute phase and causes headache, diplopia, and, in severe cases, visual loss.^4,91^ Acetazolamide, topiramate, and glucagon-like peptide-1 (GLP-1) receptor agonists can reduce production of cerebral spinal fluid and may reduce intracranial pressure and prevent vision loss.^92–94^ The efficacy and safety of these drugs in patients with CVT for the treatment of intracranial hypertension, both in the acute and chronic phase, have not been properly investigated.

### Theme 6: treatment—endovascular

#### Is there a subgroup of CVT patients who may benefit from endovascular treatment?

Rationale: The Thrombolysis or Anticoagulation for Cerebral Venous Thrombosis (TO-ACT) trial^57^ did not show benefit of endovascular treatment compared with standard medical care in patients with CVT who were at risk of poor outcome. As a result, endovascular treatment is currently only recommended as a last resort treatment in CVT patients with a malignant disease course. It is unknown, whether a subgroup of CVT patients may benefit from first-line endovascular treatment and what the optimal timing of this treatment would be. To investigate this, both clinical and imaging variables need to be evaluated to identify variables associated with favorable outcomes of endovascular treatment, similar to what has been done for patients with acute ischemic stroke.^95,96^ In addition, the outcomes of early intervention post-diagnosis need to be compared with delayed treatment triggered by clinical deterioration. When evaluating the effect of endovascular treatment for CVT, cost-effectiveness should be taken into account.

#### What is the best endovascular approach to achieve fast and safe recanalization in CVT?

Rationale: Different devices and techniques are currently used for endovascular treatment of CVT, such as mechanical thrombectomy with stent-retrievers, aspiration catheters, or balloon catheters and application of intra-sinus thrombolytic agents.^97^ It is unknown what the best approach for endovascular treatment of CVT is and the choice of device is currently at the discretion of the interventionalist who performs the procedure. All devices currently used for endovascular treatment of CVT were designed for treatment in arterial acute ischemic stroke, coronary artery disease, or peripheral vascular disease and have not been optimized for the larger diameter of sinuses involved in CVT. Therefore, a systematic evaluation of existing devices is needed. Depending on this assessment, devices specifically aimed at recanalizing the cerebral venous system may need to be developed.^97^

## Conclusion

CVT, a less common yet increasingly diagnosed stroke subtype, primarily affects young adults. Despite current best medical practices, the diagnosis is often challenging and many patients suffer from residual symptoms with substantial impact on their quality of life. The CVT summit 2023 in Amsterdam was crucial for bringing together global expertise to create a focused research agenda that targets the significant knowledge gaps in CVT.

The proposed research agenda, outlined in this article, prioritizes essential questions and areas in CVT research as viewed by the participants of the CVT summit 2023. It covers a wide range of questions, ranging from understanding of global epidemiology to improving post-CVT quality of life, developing innovative diagnostic tools, elucidating its unique pathophysiology, and evaluating novel treatment modalities. Ultimately, the goals are to guide and streamline future research efforts, promoting international and interdisciplinary collaboration, large-scale studies, and research initiatives, and facilitating necessary funding for pivotal research areas. Acquisition of funding for CVT is challenging, especially because studies generally require international collaboration and many funding opportunities are aimed at single-country studies. We believe these concerted efforts will advance our understanding of CVT and its management, and lead to improved prognosis and enhanced quality of life for CVT patients worldwide.

## Supplemental Material

sj-docx-1-wso-10.1177_17474930241242266 – Supplemental material for Reducing the global burden of cerebral venous thrombosis: An international research agenda Supplemental material, sj-docx-1-wso-10.1177_17474930241242266 for Reducing the global burden of cerebral venous thrombosis: An international research agenda by Jonathan M Coutinho, Anita van de Munckhof, Diana Aguiar de Sousa, Sven Poli, Sanjith Aaron, Antonio Arauz, Adriana B Conforto, Katarzyna Krzywicka, Sini Hiltunen, Erik Lindgren, Mayte Sánchez van Kammen, Liqi Shu, Tamam Bakchoul, Rosalie Belder, René van den Berg, Elisheva Boumans, Suzanne Cannegieter, Vanessa Cano-Nigenda, Thalia S Field, Isabel Fragata, Mirjam R Heldner, María Hernández-Pérez, Frederikus A Klok, Ronen R Leker, Lia Lucas-Neto, Jeremy Molad, Thanh N Nguyen, Dirk-Jan Saaltink, Gustavo Saposnik, Pankaj Sharma, Jan Stam, Vincent Thijs, Michiel van der Vaart, David J Werring, Diana Wong Ramos, Shadi Yaghi, Nilüfer Yeşilot, Turgut Tatlisumak, Jukka Putaala, Katarina Jood, Marcel Arnold and José M Ferro in International Journal of Stroke
