# Supplementary material for: Reducing the global burden of cerebral venous thrombosis: An international research agenda
Source: Int J Stroke. 2024 Apr 9;19(6):599–610. doi: 10.1177/17474930241242266 (PMC11292977; doi:10.1177/17474930241242266)
Supplement: sj-docx-1-wso-10.1177_17474930241242266 – Supplemental material for Reducing the global burden of cerebral venous thrombosis: An international research agenda [file sj-docx-1-wso-10.1177_17474930241242266.docx]

**SUPPLEMENTAL MATERIAL**

***Reducing the global burden of cerebral venous thrombosis: an international research agenda***

**Table of contents**

Supplemental Table 1. List of attendees of the CVT summit 2023.............................................................................2

Supplemental Table 2. Overview of panel members per research theme...................................................................4

Supplemental Figure 1. Group picture of participants in the CVT summit 2023........................................................5

**Supplemental Table 1. List of attendees of the CVT summit 2023.**

| Name | Degree | Organization | Country | Role |
| --- | --- | --- | --- | --- |
| Sanjith Aaron | MD, DM | Christian Medical College Hospital Vellore | India | Neurologist |
| Diana Aguiar de Sousa | MD, PhD | Lisbon Central University Hospital, University of Lisbon | Portugal | Neurologist |
| Antonio Arauz | MD, PhD | Instituto Nacional de Neurologia y Neurocirugia Manuel Velasco Suarez | Mexico | Neurologist |
| Marcel Arnold | MD | Inselspital Bern, University Hospital Bern | Switzerland | Neurologist |
| Tamam Bakchoul | MD, PhD | University of Tübingen | Germany | Hematologist |
| Rosalie Belder |  | Trombosestichting | The Netherlands | Representative from non-profit granting organization |
| René van den Berg | MD, PhD | Amsterdam UMC | The Netherlands | Radiologist |
| Elisheva Boumans | LLM | Patient’s representative, https://elishevaboumans.nl/ | The Netherlands | Patient’s representative |
| Suzanne Cannegieter | MD, PhD | Leiden University Medical Center | The Netherlands | Epidemiologist |
| Vanessa Cano-Nigenda | MD, PhD | Instituto Nacional de Neurologia y Neurocirugia Manuel Velasco Suarez | Mexico | Neurologist |
| Jonathan M. Coutinho | MD, PhD | Amsterdam UMC | The Netherlands | Neurologist |
| José M. Ferro | MD, PhD | Universidade de Lisboa | Portugal | Neurologist |
| Thalia S. Field | MD, MHSc | University of British Columbia | Canada | Neurologist |
| Isabel Fragata | MD, PhD | Centro Hospitalar Universitário Lisboa Central | Portugal | Neuroradiologist |
| Mirjam R. Heldner | MD, MSc | Inselspital, University hospital and University of Bern | Switzerland | Neurologist |
| María Hernández-Pérez | MD, PhD | H. Germans Trias i Pujol | Spain | Neurologist |
| Sini Hiltunen | MD, PhD | Helsinki University Hospital | Finland | Neurologist |
| Katarina Jood | MD, PhD | Sahlgrenska University Hospital | Sweden | Neurologist |
| Frederikus A. Klok | MD, PhD | Leiden University Medical Center | The Netherlands | Vascular medicine specialist |
| Katarzyna Krzywicka | MD, MPhil | Amsterdam UMC | The Netherlands | PhD student |
| Ronen R. Leker | MD | Hadassah - Hebrew University Medical Center | Israel | Neurologist |
| Ton Lensing | MD, PhD | Bayer | The Netherlands | Industry representative |
| Erik Lindgren | MD, PhD | Sahlgrenska University Hospital, University of Gothenburg | Sweden | Neurology resident |
| Lia Neto | MD, PhD | Lisbon Medical School/ North Lisbon Medical Center | Portugal | Radiologist |
| Jeremy Molad | MD | Tel Aviv Medical Center | Israel | Neurologist |
| Anita van de Munckhof | MD | Amsterdam UMC | The Netherlands | PhD student |
| Thanh N. Nguyen | MD | Boston Medical Center | United States | Interventional neurologist |
| Sven Poli | MD, PhD | University of Tübingen | Germany | Neurologist |
| Jukka Putaala | MD, PhD | Helsinki University Hospital | Finland | Neurologist |
| Dirk-Jan Saaltink | PhD | Hersenstichting | The Netherlands | Representative from non-profit granting organization |
| Mayte Sánchez van Kammen | MD, PhD | Amsterdam UMC | The Netherlands | Neurology resident |
| Gustavo Saposnik | MD, PhD | University of Toronto | Canada | Neurologist |
| Pankaj Sharma | MD, PhD | Royal Holloway University of London | United Kingdom | Neurologist |
| Liqi Shu | MD | Brown University | United States | Neurologist |
| José Souza | MD | Daiichi Sankyo | Germany | Industry representative |
| Jan Stam | MD, PhD, MA | Amsterdam UMC | The Netherlands | Neurologist |
| Turgut Tatlisumak | MD, PhD | University of Gothenburg | Sweden | Neurologist |
| Vincent Thijs | MD, PhD | Florey Institute of Neuroscience and Mental Health | Australia | Neurologist |
| David J. Werring | MD, PhD | UCL Queen Square Institute of Neurology | United Kingdom | Neurologist |
| Han Wijn |  | Medtronic | The Netherlands | Industry representative |
| Diana Wong Ramos |  | Portugal AVC- União de Sobreviventes, Familiares e Amigos | Portugal | Patient’s representative |
| Shadi Yaghi | MD, PhD | Brown University | United States | Neurologist |
| Nilüfer Yeşilot | MD | Istanbul Faculty of Medicine, Istanbul University, Istanbul, Turkey | Turkey | Neurologist |

CVT = cerebral venous thrombosis.

All attendees of the CVT summit 2023 were invited by the meeting’s organizing committee based on past involvement in CVT research. We specifically aimed to have a balanced grouped of meeting participants in terms of nationality (different continents, from different socio-economic backgrounds), male-female ratio, and years of experience.

| **Theme** | **Panel member** | **Role** |
| --- | --- | --- |
| Epidemiology and clinical features | Katarina Jood | Chair |
|  | Sanjith Aaron | Speaker |
|  | Suzanne Cannegieter | General panel member |
|  | Antonio Arauz | General panel member |
|  | Erik Lindgren | Secretary |
| Life after CVT | José M. Ferro | Chair |
|  | Thalia S. Field | Speaker |
|  | Mirjam R. Heldner | General panel member |
|  | Turgut Tatlisumak | General panel member |
|  | Sini Hiltunen | Secretary |
| Neuroimaging and diagnosis | Vincent Thijs | Chair |
|  | Diana Aguiar de Sousa | Speaker |
|  | René van den Berg | General panel member |
|  | Lia Neto | General panel member |
|  | Katarzyna Krzywicka | Secretary |
| Pathophysiology | David J. Werring | Chair |
|  | Pankaj Sharma | Speaker |
|  | Tamam Bakchoul | General panel member |
|  | Frederikus A. Klok | General panel member |
|  | Mayte Sánchez van Kammen | Secretary |
| Treatment - medication | Jonathan M. Coutinho | Chair |
|  | Jukka Putaala | Speaker |
|  | Marcel Arnold | General panel member |
|  | Shadi Yaghi | General panel member |
|  | Anita van de Munckhof | Secretary |
| Treatment – endovascular | Ronen R. Leker | Chair |
|  | René van den Berg | Speaker |
|  | Isabel Fragata | General panel member |
|  | Thanh N. Nguyen | General panel member |
|  | Liqi Shu | Secretary |

**Supplemental Table 2. Overview of panel members per research theme.**

**
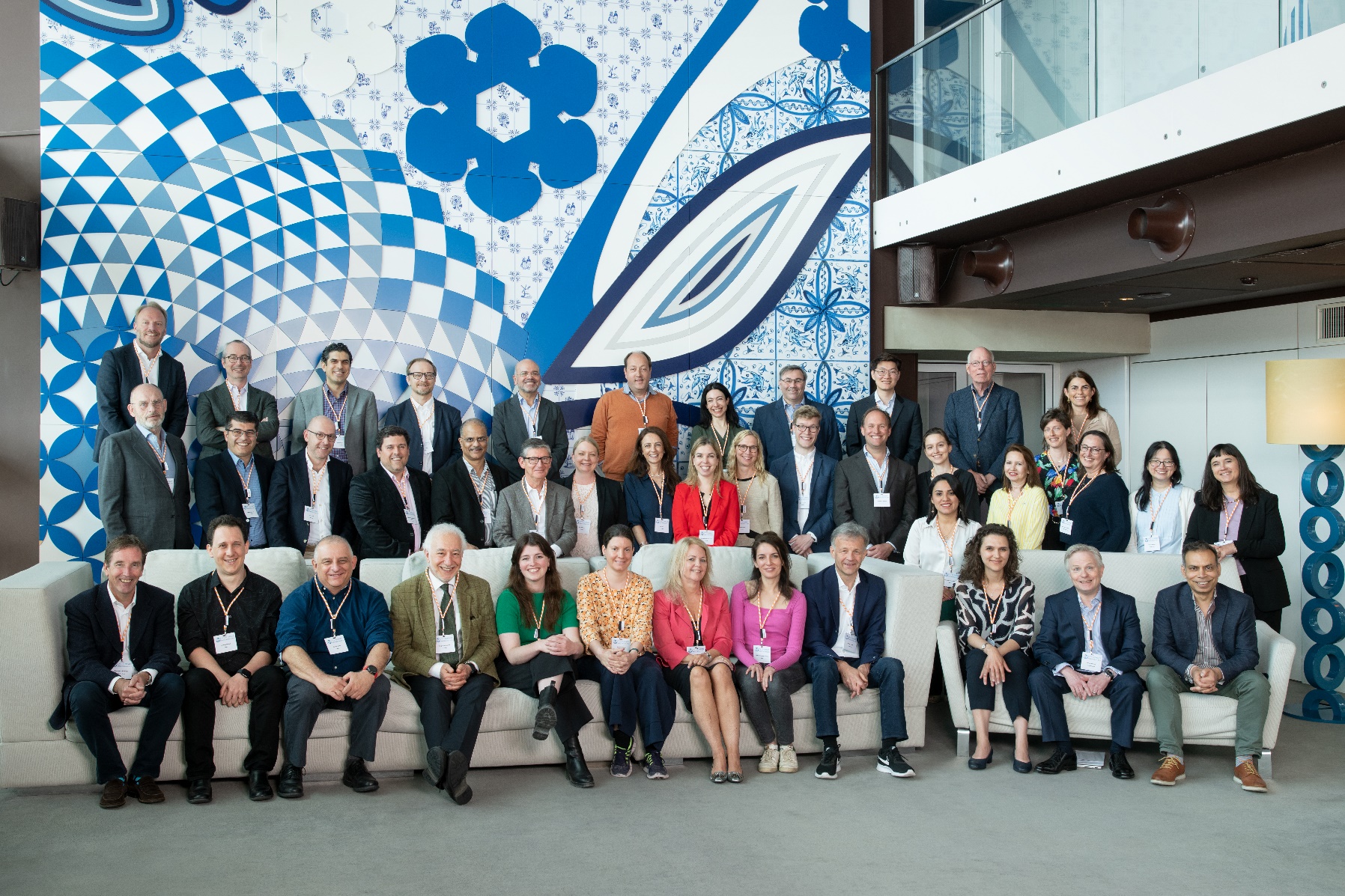
Supplemental Figure 1. Group picture of participants in the CVT summit 2023.**

CVT = cerebral venous thrombosis.
